# Phenotypic assessment of resistance in a flock of Pelibuey lambs experimentally infected with *Haemonchus contortus*

**DOI:** 10.1590/S1984-29612026022

**Published:** 2026-07-06

**Authors:** Maritza Zaragoza-Vera, Roberto González-Garduño, Oswaldo Margarito Torres-Chablé, Claudia Virginia Zaragoza-Vera, Guadalupe Arjona-Jiménez, Armando Jacinto Aguilar-Caballero, Fleider Leiser Peña-Escalona

**Affiliations:** 1 Universidad Autónoma de Yucatán, Facultad de Medicina Veterinaria y Zootecnia, Campus de Ciencias Biológicas y Agropecuarias, Mérida-Xmatkuil, Yucatán, México; 2 Universidad Autónoma Chapingo, Unidad Regional Universitaria Sursureste, Teapa, Tabasco, México; 3 Universidad Juárez Autónoma de Tabasco, División Académica de Ciencias Agropecuarias, Villahermosa, Tabasco, México; 4 Universidad Politécnica de Texcoco, Facultad de Comercio Internacional y Aduanas, Texcoco de Mora, Estado de México, México

**Keywords:** Hair sheep, nematode parasites, packed cell volume, plasma protein, resistant, susceptible, Ovinos lanados, nematódeos parasitas, volume globular, proteína plasmática, resistente, suscetível

## Abstract

The aim of this study was to determine the phenotypic variables commonly evaluated to segregate Pelibuey lambs as resistant or susceptible to *Haemonchus contortus* infection. Twenty-one lambs were infected over 3 consecutive weeks with 200 larvae per kg of body weight. The lambs were dewormed 42 days after the first infection. A second infection was performed during the growth stage (32 weeks of age), and a third infection at 1 year of age. After the first infection, the lambs were segregated as resistant (<761 eggs per gram of feces, EPG) or susceptible (>761 EPG). Packed cell volume (PCV) and total plasma protein (TPPr) during the first stage were lower (25.14% and 6.56 g/dL) than during the second stage (30.06% and 7.1 g/dL, respectively). A notable decrease in EPG values in both groups occurred from the first to the second experimental infection (resistant: 510 ± 663 to 26 ± 83 EPG; susceptible: 3621 ± 6246 to 76 ± 361 EPG). Pelibuey lambs can be segregated as susceptible or resistant to *H. contortus* infection from 5 months of age following the first infection. The most informative phenotypic markers for segregation identified in the present study were EPG, PCV, and TPPr.

## Resumo

O objetivo foi identificar as variáveis fenotípicas comumente avaliadas para classificar cordeiros Pelibuey em resistentes (RES) ou suscetíveis (SUS) à infecção por *Haemonchus contortus*. Vinte e um cordeiros foram infectados durante três semanas com 200 larvas por kg de peso corporal (PC). Os cordeiros foram vermifugados 42 dias após a primeira infecção. Uma segunda infecção foi realizada (32 semanas de idade) e uma terceira infecção com um ano de idade. Após a primeira infecção, os cordeiros foram classificados como RES (< 761 ovos por grama de fezes, OPG) ou SUS (> 761 OPG) à infecção. O volume globular (VG) e a proteína plasmática total (PPT) durante o primeiro estágio foram menores (25,14% e 6,56 g/dL) do que no segundo (30,06% e 7,1 g/dL, respectivamente). Observou-se uma diminuição notável nos valores de OPG em ambos os grupos (RES de 510 para 26 OPG e SUS de 3621 para 76 OPG) da primeira para a segunda infecção experimental. Cordeiros Pelibuey podem ser classificados como suscetíveis ou resistentes à infecção por *H. contortus* a partir dos cinco meses de idade após a exposição à primeira infecção. Os melhores marcadores fenotípicos para a classificação dos cordeiros encontrados neste estudo foram OPG, VG e PPT.

## Introduction

Gastrointestinal nematodes (GINs) represent one of the principal constraints to sheep production in tropical regions (Ilangopathy et al., 2019). In grazing-based systems, *Haemonchus contortus* is widely recognized as the most pathogenic species, inducing severe infections characterized by marked anemia and, in some cases, mortality, particularly among lambs, which constitute the most susceptible group within the flock (Berton et al., 2019; Casanova et al., 2018).

Anthelmintic drugs have been extensively used over the past decades to control GIN infections and prevent adverse effects on animal health. However, the repeated and indiscriminate application of these compounds has driven the widespread emergence of anthelmintic resistance among GIN populations (Claerebout & Geldhof, 2020; Dey et al., 2020; Pawar et al., 2019). Additionally, some anthelmintic drugs have caused environmental damage, particularly affecting dung beetle populations (Martínez et al., 2018; Verdú et al., 2020). Consequently, sustainable control alternatives have been explored, including the selection of breeds with enhanced resistance to GINs, such as Pelibuey sheep (Díaz-Rivera et al., 2000). Pelibuey ewes are widely raised in the humid tropics of Mexico, where this breed has been characterized by its adaptation to warm, humid environments, low-quality forage, and tolerance to parasitic infections (Aguirre-Serrano et al., 2020; Hinojosa-Cuéllar et al., 2015; Luna-Palomera et al., 2019).

Resistance to GINs is expressed when animals are able to limit parasite establishment and reduce fecal nematode egg output (Toscano et al., 2019). The selection of resistant lambs has been assessed using phenotypic markers such as fecal egg count (FEC), packed cell volume (PCV), total plasma protein (TPPr), peripheral eosinophil counts (PEC), body condition score, and changes in live weight (Ojeda-Robertos et al., 2017; Palomo-Couoh et al., 2017; Zaragoza-Vera et al., 2023b).

Although Pelibuey sheep are considered relatively resistant to GINs during pregnancy, animals may exhibit increased susceptibility to infection at certain physiological stages, such as weaning, growth, and lactation (González-Garduño et al., 2018). Identifying host responses to infection is therefore essential for the early selection of resistant individuals. The proposed hypothesis is that Pelibuey lambs exhibit differential GIN egg counts during the first post-weaning infection according to their level of resistance or susceptibility. Therefore, animals can be selected based on their innate resistance, as reflected by lower FEC. In subsequent infections, lambs develop acquired resistance, leading to a progressive reduction in FEC with repeated exposure. Accordingly, the aim of this study was to evaluate phenotypic variables in Pelibuey lambs classified as resistant or susceptible to *H. contortus* infection across three growth stages under humid tropical conditions. Specifically, the objective was to determine the parasitological differences between resistant and susceptible Pelibuey lambs post-weaning in successive *H. contortus* infections in order to identify the optimal time for selecting lambs.

## Material and Methods

### Study area

This study was conducted between July 2019 and April 2020 on a commercial farm in Salto de Agua, Chiapas, Mexico (17°33′00″ N, 92°20′00″ W). The climate of the region is warm and humid, with rainfall throughout the year, a mean annual temperature of 28.6 °C, and an average annual precipitation of 3,369.5 mm (SMN, 2026).

### Management of sheep

A cohort of 21 contemporary lambs, from a flock of 60 ewes previously evaluated and classified as having high genetic resistance to *H. contortus* infection (Zaragoza-Vera et al., 2023a), was included in the study. The lambs were born between 1 February and 18 March at the Center for Training and Reproduction of Minor Species (CECAREM).

### Pre-infection stage

The lambs were maintained under confinement and free of GIN infections until weaning. During this period, they were fed commercial concentrate, dam’s milk, and water *ad libitum*, and were housed in raised-floor pens. Weaning was performed at 90 days of age; at this time, blood samples were collected and body weight (BW) was recorded. In addition, fecal samples were collected directly from the rectum of each lamb using polyethylene bags and subsequently transported to the laboratory to confirm the absence of gastrointestinal parasites using the modified McMaster technique (Rodríguez-Vivas, 2015) with a sensitivity of 50 eggs per gram of feces (EPG).

Infective third-stage larvae (L_3_) of *H. contortus* were donated by the Laboratory of Helminthology at the National Centre for Disciplinary Research in Animal Health and Innocuity (CENID-SAI), National Institute for Research in Forestry, Agriculture, and Livestock (INIFAP). After two donor lambs were established with monospecific infections induced by oral administration of 300 L_3_ per kg of BW, larvae were cultured following methodologies previously described (Zaragoza-Vera et al., 2023b). These larvae were used to induce experimental infection throughout study, and mono-infection with *H. contortus* was confirmed by copro-cultures at all stages of the study.

### First infection

The first experimental infection was performed during the post-weaning stage, when lambs were 5 months old. Infection was induced orally on days 1, 7, and 14 with 200 L_3_ of *H. contortus* per kg of BW. Infection establishment was monitored on days 21, 28, 35, and 42 post-infection by collecting and examining fecal samples using the modified McMaster technique. In parallel, hematological changes were evaluated through blood sampling and determination of PCV, PEC, and TPPr concentrations.

Subsequently, the lambs were dewormed 42 days after the first infection using levamisole at a dose of 7.5 mg/kg BW (Helmicin^®^ 12%; Sanfer, Mexico). After treatment, the lambs were maintained under confinement for a 4-week inter-infection period, after which the second experimental infection was performed (Table 1).

**Table 1:** Age of lambs and study stages on the body weight and the final number of infective larvae (L_3_) per lamb in each infection.

**Stage and infection**	**Age (weeks old)**	**Weight (Kg)**	**Treatment**
Donors (independent)	12	15.8 ± 1.9	300 L_3_/kg BW
Pre-infection	12-19	16.7 ± 1.7	No infection
First infection	20,21,22	18.65± 2.9	200 L_3_/kg BW per week
Deworming	26	24.59 ± 3.6	Levamisole
Second infection	30,31,32	25.96± 3.2	200 L_3_/kg BW per week
Deworming	36	26.25± 3.3	Levamisole
Third infection	50,51,52	27.07± 3.9	200 L_3_/kg BW per week

### Second infection

**Figure 1:**
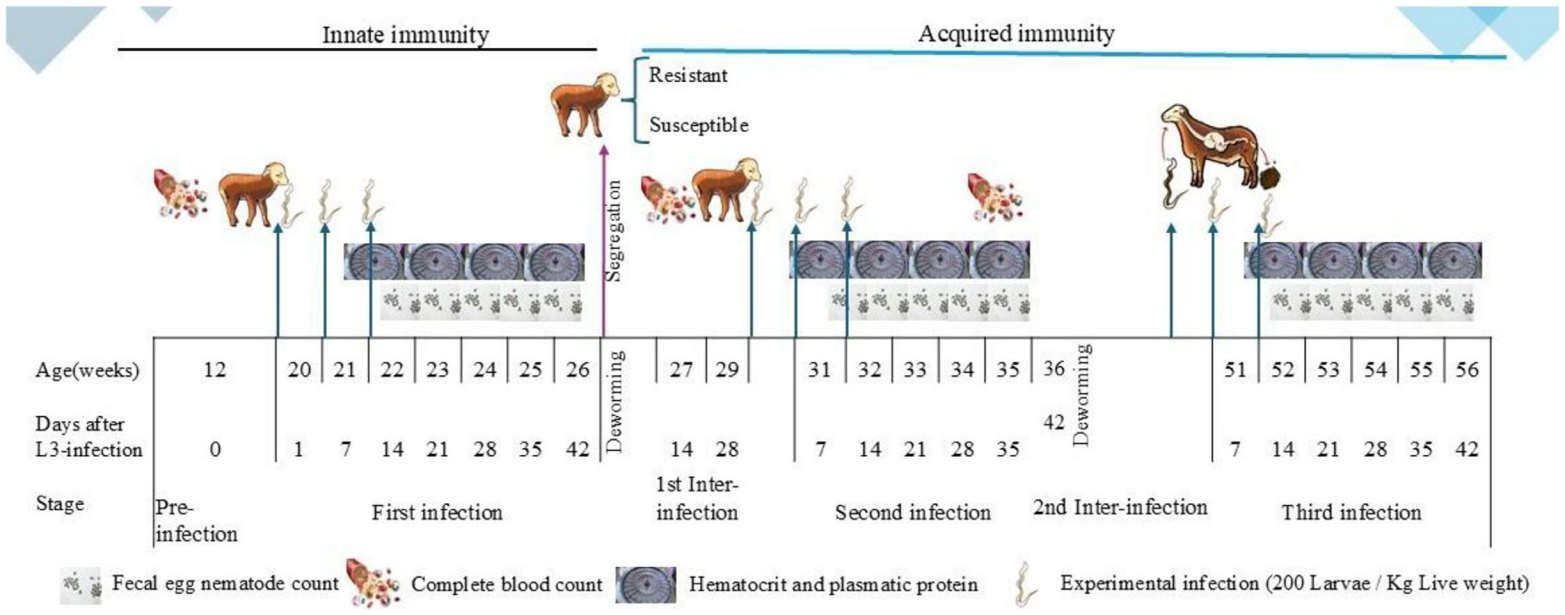
Schematic representation of the experimental infections of Pelibuey lambs with *Haemonchus contortus* infective larvae for the development of acquired resistance.

The second infection was performed during the growth stage, when the lambs were 7.5 months of age. Infection was induced under the same conditions described for the first infection. Likewise, infection establishment was monitored by evaluating fecal and blood samples on days 21, 28, 35, and 42 post-infection (Figure 1). The lambs were dewormed 42 days after the second infection using levamisole at a dose of 7.5 mg/kg BW (Helmicin^®^ 12%; Sanfer).

### Third infection

The third experimental infection was performed when the animals reached 1 year of age. Infection was induced under the same conditions previously described. However, a low level of infection was established 42 days post-infection, and consequently, no anthelmintic treatment was administered at the end of the study.

The number of observations obtained in each stage was consistent with the type of samples. Feces samples were collected over eight weeks in the first stage, seven weeks in the second stage, and six weeks in the third stage, resulting in a total number of samples of 168, 147, and 116 in the respective stages.

### Body weight (BW)

The lambs were weighed weekly using a digital hanging scale (Rhino^®^, model BAC300; Mexico) with a precision of 100 g, and all measurements were recorded. BW change was calculated as the difference between the initial and final weights within each experimental stage.

### PCV, PEC, and TPPr

Whole blood samples were collected from each animal by jugular venipuncture into EDTA tubes (Vacutainer^®^; BD Biosciences, Franklin Lakes, NJ, USA) for the determination of PCV and PEC. PCV (%) was measured using the microhematocrit technique, whereas PEC was determined using a Neubauer chamber (cells × 10^3^/µL) after dilution with Carpentier solution (Dawkins et al., 1989). TPPr concentrations were measured using a refractometer (Master-Vet; ATAGO, Tokyo, Japan).

### FEC determination

Fecal samples were collected during the pre-infection stage to confirm the absence of GINs and subsequently throughout the study during the experimental infections, as schematized in Figure 1. All fecal samples were processed using the McMaster technique to determine the number of EPG (Rodríguez-Vivas, 2015). At the end of the first experimental stage, EPG values were used to classify lambs as susceptible or resistant to *H. contortus* infection. The overall mean EPG count minus two standard errors was used as the cut-off point for classification, as described by Palomo-Couoh et al. (2016).

### Statistical analysis

Parasitological, hematological, and productive data were analyzed using a repeated-measures model over time. FEC data were log-transformed [log (EPG + 1)] to stabilize variance and approximate a normal distribution (Rodríguez et al., 2015). Three growth stages were included in the analysis as sources of variation, and statistical modeling was performed using the MIXED procedure of SAS (version 9.4, 2017; SAS Institute Inc., Cary, NC, USA) according to the following model:


$$Yijkl\;=\;\mu \;+\;\delta i\;+\;\zeta j\;+\;\delta \zeta ij\;+\varphi k\left(ij\right)\;+\;\psi l\;+\;\delta \psi il\;+\;\zeta \psi jl\;+\;\delta \zeta \psi ijl\;+\;\varepsilon ijkl$$ (1)

*Y*_ijkl =_ response variable (parasitological, hematological, and productive parameters); μ = general mean; δ_i_ = effect of infection (i = first, second, and third infection); ζ_j_ = effect of type of lamb (j = resistant or susceptible); δζ_ij_ = interaction of infection and type of lamb; Φ_k(ij)_ = random effect associated with the k-th animal (subject) within the ij-th treatment; ψ_l_ = effect of time or sampling (l = 1, 7, 14, 21, 35, and 42 days); δψ_il_ = interaction of infection and time; ζψ_jl_ = interaction of type of lamb and time; δζψ_ijl_ = interaction of infection, type of lamb, and time; ε_ijkl_ ∼ N (0, σ^2^_e_) = residual error.

Least-squares means of parasitological and hematological variables for susceptible and resistant groups within each experimental stage were compared using Tukey’s post hoc test to detect statistically significant differences (P < 0.05). Pearson correlations between hematological and immunological variables were also performed using SAS (2017) at each stage, considering the categories of resistant and susceptible lambs.

## Results

### Effect of first infection

During the first infection, higher FEC (2784 ± 4745 EPG) was associated with reduced PCV, reflecting the impact of infection, and low PEC was observed in lambs after weaning (Table 2). After deworming, the average egg excretion was 13.8 EPG, and during the inter-infection stage, counts dropped to zero.

**Table 2:** Variable response of Pelibuey lambs during the first experimental infection with *H. contortus*.

Experimental stages	**Body weight** (Kg)	**FEC** (EPG)	**TPPr** (g/dL)	**PCV** (%)	**PEC** (cell x 10^3^/µl)
First infection (pre-segregation)	18.64	2784	6.56	25.14	0.104
Standard deviation (SD)	6.18	4745	0.79	6.79	0.13
Standard error	1.32	1012	0.07	0.60	0.01

FEC: Fecal egg counts (Eggs per gram of feces EPG). TPPr: Total plasmatic proteins (g/dL). PCV: Packed cell volume (%). PEC: Peripheral eosinophil count (cell x 10^3^/µl).

### Segregation (first infection)

The first experimental infection was used to classify lambs as resistant (n = 6) or susceptible (n = 15). The cut-off value was set at 761 EPG, calculated from the overall mean EPG (2784) minus twice the standard error (1011.5 EPG), separating the animals into two groups (2784 − 2023 = 761 EPG). Egg excretion was markedly higher in susceptible lambs than in resistant lambs during the first stage of infection (Table 3), which was accompanied by lower PCV and TPPr values in susceptible animals. By contrast, no statistically significant differences were detected in eosinophil counts or live weight between groups (P > 0.05).

**Table 3:** Phenotypic responses (mean ± SD) of post-weaning lambs classified as resistant or susceptible to *Haemonchus contortus* infection at three stages.

**Experimental stage**	**N**	**FEC** (EPG)	**TPPr** (g/dL)	**PCV** (%)	**PEC** (cell x 10^3^/µl)	**BW** (Kg)
**Resistant lambs (n=6)**						
Stage 1: Post-weaning	48 (8)	510±663^b^	6.99±0.53^a^	27.5±5.6^b^	0.10±0.12^b^	17.6±3.4^b^
Stage 2: Growth	42 (7)	26±83^c^	7.07±0.53^a^	31.3±3.6^a^	0.26±0.14^a^	25.0±2.3^a^
Stage 3: Yearling	36 (6)	37±102^c^	5.97±0.65^c^	30.1±3.8^a^	0.25±0.18^a^	25.9±1.3^a^
**Susceptible lambs (n=15)**						
Stage 1: Post-weaning	120 (8)	3621±6246^a^	6.40±0.82^b^	24.3±7.0^c^	0.10±0.14^b^	19.0±5.5^b^
Stage 2: Growth	105 (7)	76±361^c^	7.12±0.55^a^	29.5±4.1^a^	0.26±0.18^a^	26.4±7.4^a^
Stage 3: Yearling	90 (6)	25±65^c^	6.04±0.76^c^	29.0±4.6^a^	0.28±0.21^a^	27.6±7.9^a^

SD: Standard deviation. N: Number of total observations (study weeks). FEC: Fecal egg nematode count. EPG: Eggs per gram of feces. TPPr: Total plasmatic proteins. PCV: Packed cell volume. CEP: Peripheral eosinophil count. Different letters in the same column indicate statistically significant differences (P<0.05). N: Number of observations (samples) by each lamb type and infection stage. Samples from the prepatent period were not taken into account for statistical analysis.

### Acquired resistance (second and third infections)

The second and third experimental infections resulted in lower FEC in both resistant and susceptible lambs despite the use of a trickle infection, suggesting the development of acquired resistance during the first experimental infection. The most notable finding was the increase in PEC compared with the first stage, indicating cellular immune development. In the case of TPPr, humoral immune development was observed during the second infection, reflected in higher TPPr values in both groups, followed by a reduction in 1-year-old animals. An increase in PCV was also observed during the second and third experimental infections (Table 2).

The lowest BW was observed during the first infection, showing differences compared with subsequent stages. However, no differences between the two groups of lambs were found at any stage (P > 0.05).

### Variable response over time

**Figure 2:**
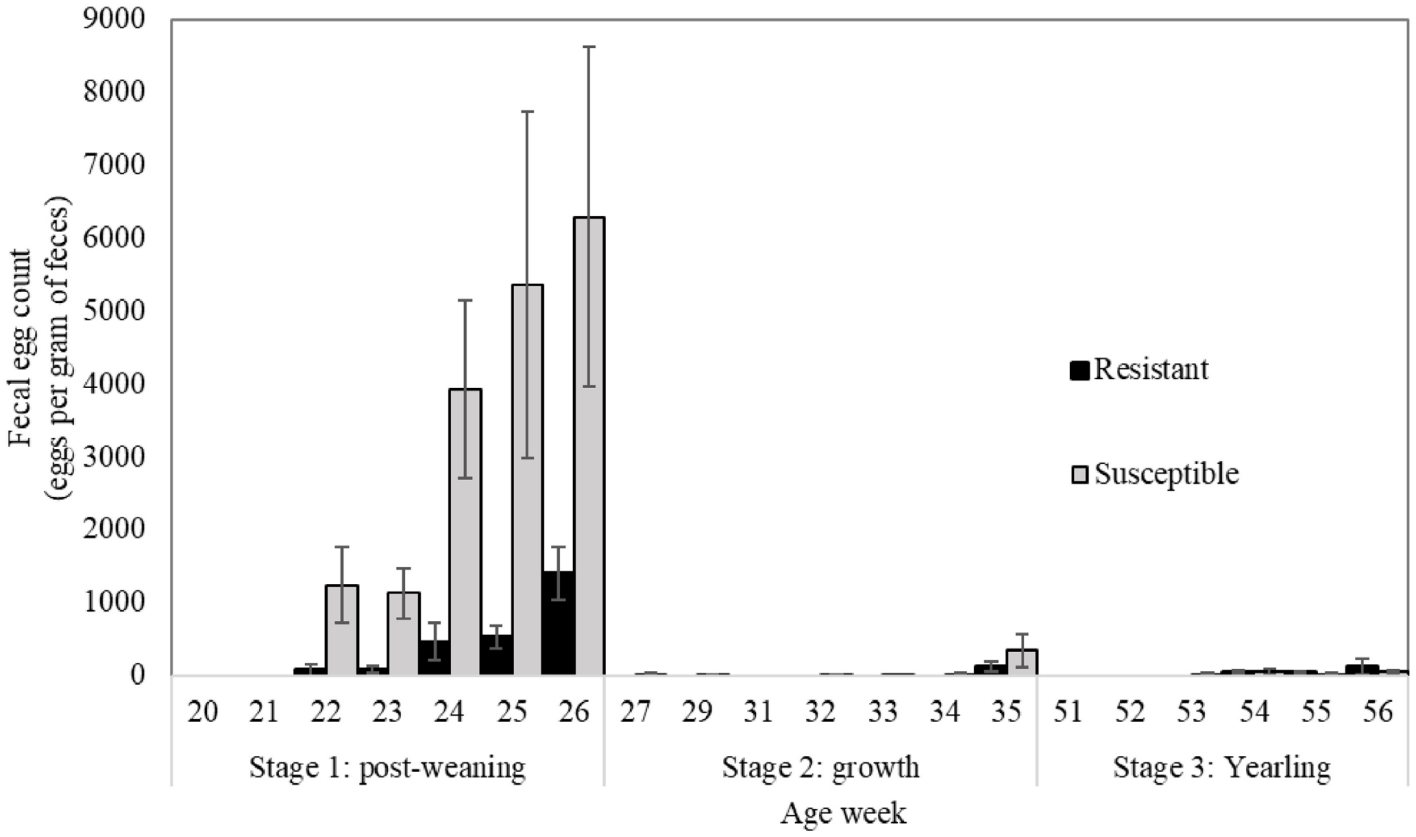
Fecal egg count (FEC) of *H. contortus* during three stages with experimental infection in Pelibuey lambs. Bars on each sample day represent standard error.

Susceptible lambs reached a maximum egg excretion of 6293 ± 2328 EPG, whereas resistant lambs exhibited 1400 ± 367 EPG during the first infection (Figure 2). By contrast, during the second infection, a marked reduction in FEC was observed in both resistant and susceptible groups, with values below 340 EPG in susceptible lambs and below 140 EPG in resistant lambs. During the third experimental infection, both groups showed similar patterns (P > 0.05), characterized by the lowest levels of *H. contortus* egg excretion.

**Figure 3:**
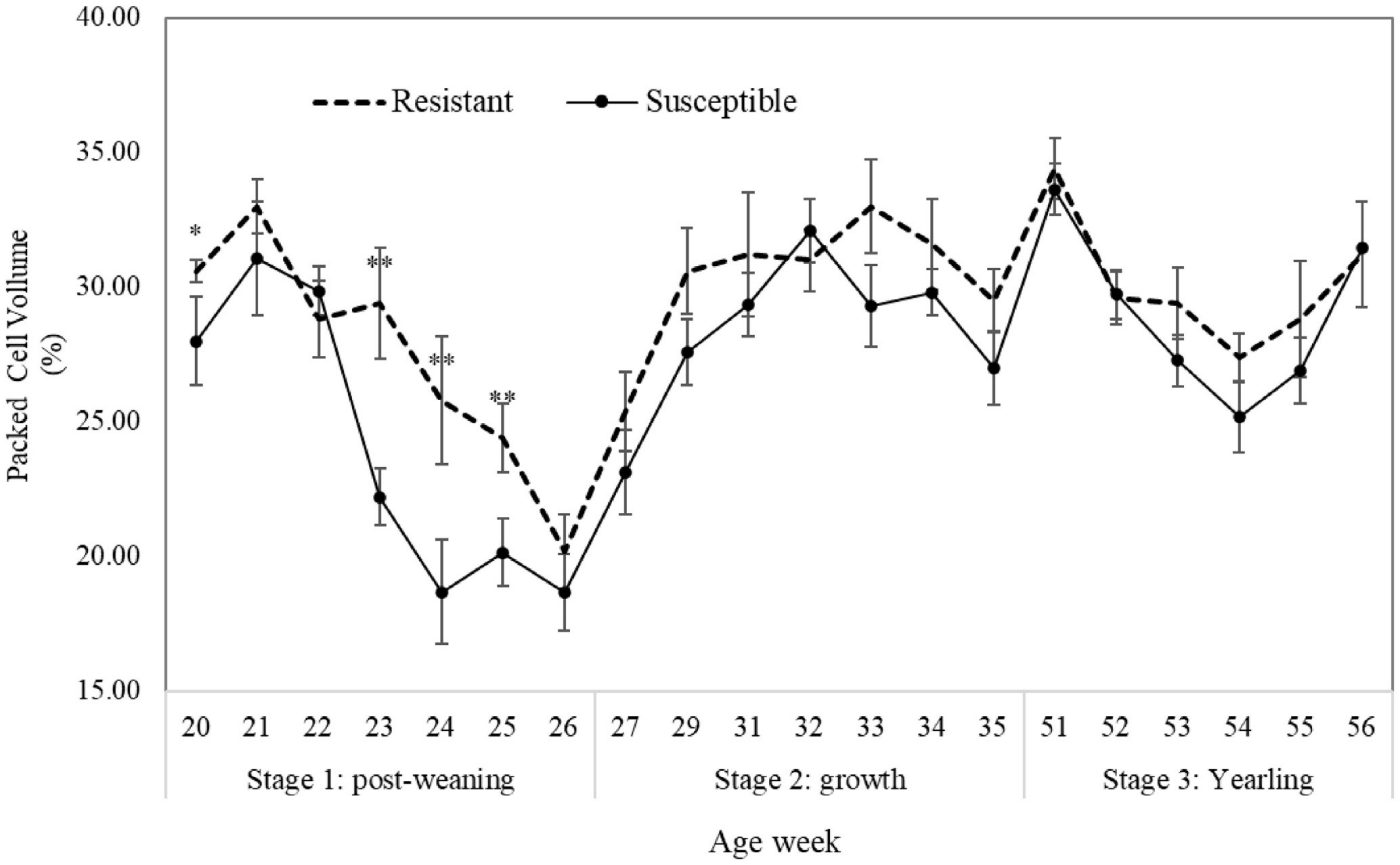
Packed cell volume (PCV) during three experimental infection stages in Pelibuey lambs. *Significant differences (P < 0.05); **Highly significant differences (P < 0.01). Bars on each sample day represent standard deviation.

Susceptible lambs showed a marked decline in PCV at 24 days post-infection during the first infection, approaching the anemia threshold (24%). Recovery was observed in both susceptible and resistant groups during the second infection. During the third infection, a reduction in PCV was recorded at 54 weeks of age; however, values did not fall to anemic levels and subsequently increased to approximately 30% (Figure 3).

**Figure 4:**
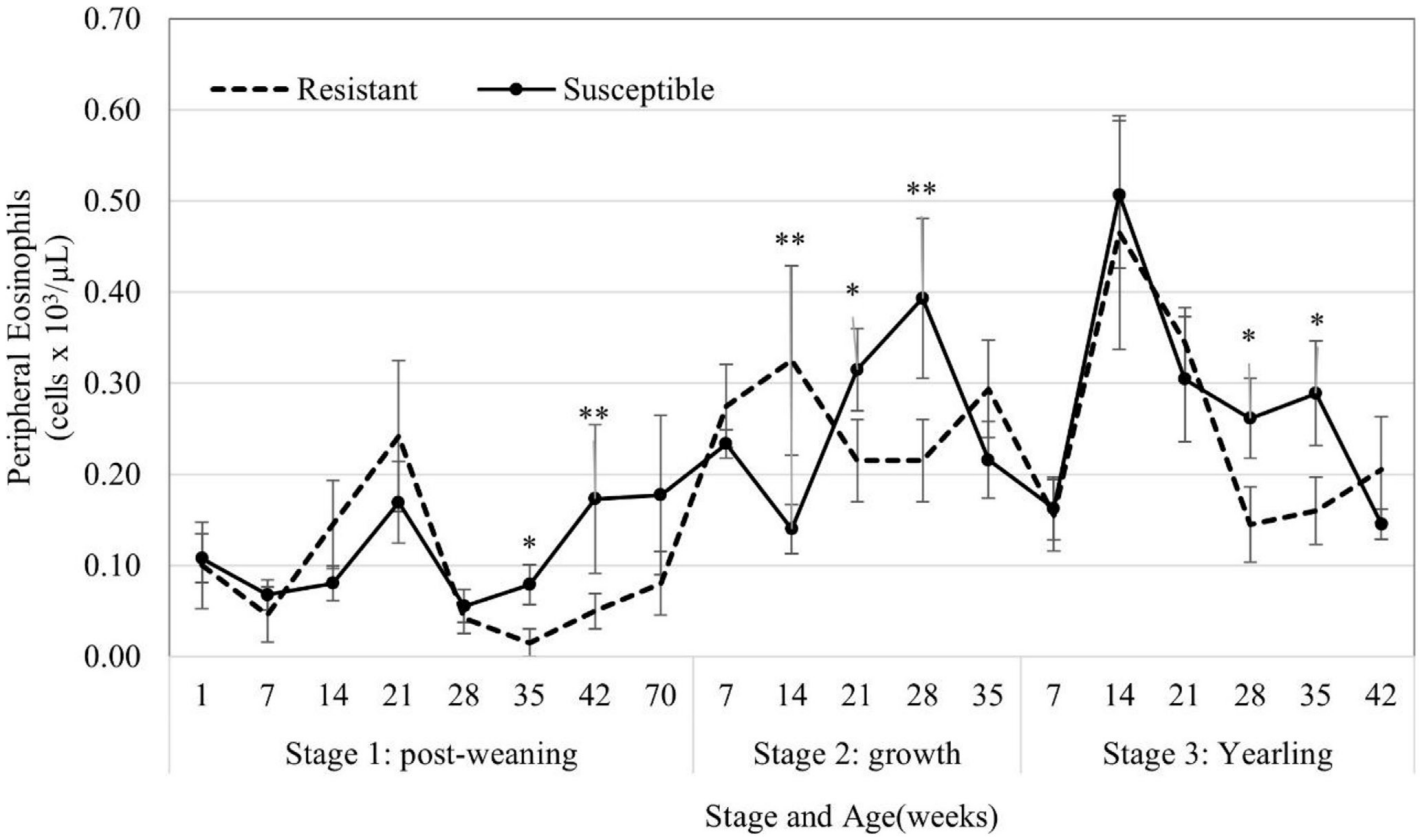
Peripheral eosinophil counts during three experimental infection stages in Pelibuey lambs. *Significant differences (P < 0.05); **Highly significant differences (P < 0.01). Bars on each sample day represent standard deviation.

Across the three experimental infections, an increase in PEC was recorded. During the first infection, both groups showed increased counts at 21 days post-infection, whereas during the second infection, resistant lambs exhibited an earlier increase at 14 days, while susceptible lambs peaked at 28 days. In the third infection, similar temporal patterns were observed; however, susceptible animals maintained elevated counts up to 35 days, whereas resistant lambs showed a reduction by 28 days post-infection (Figure 4).

In both resistant and susceptible lambs, EPG showed negative correlations with TPPr and PCV; however, these associations were consistently stronger in susceptible animals. A strong positive correlation between EPG and PEC at 28 and 35 days post-infection was observed only in resistant lambs, whereas no significant association between these variables was detected in susceptible lambs. In addition, a negative correlation between EPG and live weight was evident only in susceptible lambs. Higher positive correlations between TPPr and PCV were also recorded in susceptible lambs compared with resistant lambs (Table 4).

**Table 4:** Correlation coefficients of resistant and susceptible Pelibuey lambs in the first infection with gastrointestinal nematodes.

Days post infection	Resistant					Susceptible				
	14	21	28	35	42	14	21	28	35	42
EPG x TPPr		-0.89**	-0.39	-0.01	-0.63*		-0.63**	-0.75**	-0.56**	-0.60**
EPG x PCV		-0.41	-0.52*	-0.26	-0.07		-0.29	-0.47*	-0.40	-0.51*
EPG x PEC		-0.58	0.81**	0.75*	-0.66*		-0.32	0.06	-0.03	-0.11
EPG x BW		-0.50	0.46	0.22	0.35		-0.47*	-0.60**	-0.68**	-0.69**
TPPr x PCV	0.49	0.22	0.42	0.62^*^	0.01	0.10	0.43*	0.73**	0.54**	0.85**
TPPr x PEC	0.42	0.33	-0.10	0.60	0.29	-0.17	-0.09	0.00	0.16	0.30
TPPr x BW	0.72*	-0.33	-0.91^**^	-0.30	-0.39	0.47*	0.12	0.24	0.19	0.63**
PCV x PEC	0.07	0.75*	-0.08	0.33	-0.67	-0.22	0.03	-0.18	-0.12	0.24
PCV x BW	0.59	0.29	-0.27	-0.55	0.56	-0.09	0.10	0.15	0.43	0.52*
PEC x BW	0.21	0.34	0.38	-0.23	-0.52	0.26	0.02	0.16	-0.28	0.25

EPG: Eggs per gram of feces, PEC: Peripheral eosinophils (cell x 10^3^/µl), PCV: Packed cell volume (%), TPPr: Total plasma protein (g/dL), BW: Body weight (Kg).

* Significant correlation (P<0.05);

** Highly significant correlation (P<0.01).

## Discussion

Pelibuey sheep are considered one of the most important breeds in Mexican production systems because of their adaptability to tropical environments, including their relative resistance to GINs (Maza-Lopez et al., 2020; Zaragoza-Vera et al., 2023a). However, the immunological mechanisms underlying resistance to GINs and their relationships with phenotypic variables remain poorly understood. Therefore, the objective of this study was to characterize the performance of commonly evaluated phenotypic traits used to segregate hair sheep as resistant or susceptible to gastrointestinal parasite infections.

The phenotypic variables measured in the present study, evaluated without prior segregation of lambs (Table 2), allowed the overall effects of the experimental infections on these traits to be assessed. FEC has long been the most widely used criterion for classifying lambs as resistant or susceptible (Hayward, 2022; McRae et al., 2014; Sallé et al., 2021) in both wool and hair breeds, including Blackbelly, Katahdin, and Pelibuey (Muñoz-Guzmán et al., 2006; Palomo-Couoh et al., 2017).

The innate resistance of the flock evaluated in this study was reflected during the first experimental infection (Mcrae et al., 2015), in which the overall average FEC of the flock reached 2784 ± 4745 EPG, and lambs were subsequently segregated as resistant (<761 EPG) or susceptible (≥761 EPG). The FEC value recorded here was lower than those reported for wool breeds such as Dorset × Rambouillet × Finnsheep (3,647 ± 770 EPG). However, Pelibuey lambs exhibited higher FEC than St. Croix sheep (1,280 ± 867 EPG), a breed also considered resistant (MacKinnon et al., 2015). Similar ranges have previously been reported for Pelibuey sheep (2400 to 2800 EPG) (Morteo-Gómez et al., 2004). Collectively, these findings support previous reports indicating that Pelibuey sheep show greater resistance than Blackbelly, Katahdin, and Dorper breeds, which predominate in tropical production systems in Mexico (Palomo-Couoh et al., 2017; Zaragoza-Vera et al., 2019).

Although the total sample size was limited (n = 21) and the number of resistant lambs was small (n = 6), similar percentages of resistant lambs have been obtained in previous studies that classify lambs according to EPG (González Garduño et al., 2019). However, this constitutes a limitation of the present study because obtaining conclusive results for highly variable parameters such as PEC would require a larger sample size to allow valid comparisons between resistant and susceptible groups.

The development of acquired resistance to GINs in the Pelibuey breed was supported by the marked reduction in flock FEC observed after the first experimental infection, which declined from 2784 EPG in the first stage to 60 and 29 EPG in the second and third stages, respectively, in agreement with previous reports (González Garduño et al., 2019). Another variable associated with resistance was PCV. During the first infection, PCV decreased from normal values (approximately 30%) to below the anemia threshold (24%). By contrast, PCV was not significantly affected during the second and third infections, with mean values of 30 and 29.4%, respectively. Although the initial PCV values served as a reference to assessing changes during infection, this study was limited by the absence of a control group (uninfected lambs) with which to compare the magnitude of changes over time in infected versus uninfected animals. This variable is widely used when *H. contortus* predominates in sheep; however, it is not routinely recorded by producers because blood sampling is required (Cunha et al., 2024).

During the third experimental infection, both groups of lambs were able to control *H. contortus* infection, and the low EPG levels precluded discrimination between susceptible and resistant animals. For this reason, selection should be performed during the first infection, when lambs express innate resistance, because responses to subsequent challenges may compromise classification if segregation is conducted at later stages. These findings are likely related to the adaptive capacity of the Pelibuey breed to regulate GIN infections. Selection is important because it helps reduce parasitic burden at other stages, such as the peripartum period, during which ewes experience a reduction in immunity and become more susceptible; this is also a stage at which the selection of resistant sheep can be carried out (Pereira et al., 2020).

Across the three experimental infections, eosinophil counts increased between the third and fourth weeks post-infection; however, mean PEC during the first infection (0.104 × 10^3^ cells/µL) was lower than that recorded during the second (0.262 × 10^3^ cells/µL) and third infections (0.269 × 10^3^ cells/µL). A comparable pattern was reported in experimentally infected Blackbelly and Columbia sheep, in which eosinophil counts increased significantly between the fifth and ninth weeks relative to non-infected controls (Muñoz-Guzmán et al., 2006). Similar responses have also been described in Blackbelly sheep subjected to successive infections, where reinfection elicited higher eosinophil counts, consistent with the present findings and within comparable ranges (Terefe et al., 2007).

BW is a variable that has been used as an indicator of nematode resistance associated with productive parameters, especially in lines selected for wool, although correlations with BW have generally shown negative or near-zero values (Ferreira et al., 2021). This variable may be misleading because it is strongly influenced by normal growth and, in this case, by the small sample size; therefore, significant differences were detected only between the first experimental infection and subsequent stages. TPPr values fluctuated across the three stages of the study. This variable reflects the combined concentrations of albumin and globulins in blood, and elevated values may indicate increased immunoglobulin production, as observed during the second infection. By contrast, TPPr reached its lowest levels during the third infection. This variable was also limited by the lack of a control group to compare TPPr performance between infected and uninfected animals. Lower TPPr values have also been reported in susceptible Lanada Creole sheep during the postpartum period (Pereira et al., 2020). Resistant sheep are capable of mounting a sustained immune response, as supported by the negative correlation between parasite burden and serum globulin concentrations (Muñoz-Guzmán et al., 2006), which in the present study was reflected by the inverse relationship between EPG and TPPr.

### Effect of segregation

The data obtained in this study indicate that Pelibuey lambs can be segregated into resistant or susceptible categories from approximately 5 months of age, following the first experimental infection. Resistance to GINs in sheep is defined as the capacity to prevent ingested infective larvae from establishing or developing into adult worms, thereby reducing FEC and maintaining more favorable health indicators than those observed in susceptible animals. Variables commonly used to assess resistance include nematode mortality and fecundity, FEC, PCV, eosinophilia, and immunoglobulin levels (e.g., IgA, IgM, and IgG) (Cunha et al., 2024). Under the conditions of the present study, the most informative phenotypic variables for segregating Pelibuey lambs were EPG, PCV, and TPPr. By contrast, LW and PEC were not reliable variables for classification.

In Brazilian Somali crossbreed sheep, another breed considered resistant, mean EPG values in resistant lambs were similar to those observed in the present study (510 vs. 508 EPG). However, susceptible Pelibuey lambs exhibited higher mean EPG than Somali sheep (3621 vs. 1311 EPG). Nevertheless, at 35 days after the first infection, the maximum FEC recorded in Pelibuey lambs was lower than that observed in Somali sheep (1500 vs. 2963 EPG) (Zaros et al., 2014). Despite these differences, the dynamics of FEC were broadly similar between the two studies.

During the first infection, PCV and TPPr values in Pelibuey lambs were comparable to those reported for Brazilian Somali sheep, in which resistant animals exhibited PCV values of 27.2% and susceptible animals 20.5%, whereas in the present study, resistant Pelibuey lambs showed 27.5% and susceptible lambs 24.3%, respectively. In other studies, susceptible animals typically present hypoproteinemia, anemia, diarrhea, and inappetence, which constitute the main clinical signs associated with GIN infections (Zaros et al., 2014). Compared with Pelibuey lambs without infection, both values were lower, as normal PCV values are close to 39% (Montero-de los Santos et al., 2024).

Post-infection eosinophilia has been documented in Pelibuey and Katahdin lambs, although interbreed differences have been reported, potentially reflecting variation in resistance to GINs (Palomo-Couoh et al., 2017), Nevertheless, PEC did not differ significantly between resistant and susceptible animals in Somali sheep (745–768 cells/µL) (Zaros et al., 2014) or in Pelibuey lambs (1000 cells/µL). Although eosinophils are recognized as important effector cells in protective responses against GIN infections in sheep (Balic et al., 2002; Robinson et al., 2010), their role appears to be more pronounced in acquired immunity, as evidenced by the increase in PEC from the first to the second experimental infection observed in the present study.

Similarly to the present findings, no differences in BW between resistant (22.5 kg) and susceptible (21.3 kg) animals were reported throughout the experimental period in another study (Zaros et al., 2014). The high variability in parasitological responses among Pelibuey lambs resulted in fluctuating correlation coefficients across infection stages. Nevertheless, consistent patterns, such as negative correlations between EPG and both PCV and TPPr, have also been documented in this breed and in Katahdin lambs (Palomo-Couoh et al., 2017). Positive associations between hematological and immunological variables have likewise been described, including correlations between total lymphocyte and eosinophil counts.

## Conclusion

The data obtained in this study indicate that Pelibuey lambs can be classified as susceptible or resistant to *H. contortus* infection after weaning, following their first experimental challenge. Under the conditions evaluated, the most informative phenotypic markers for segregation were EPG, PCV, and TPPr. Selection of resistant lambs should be performed after weaning, following the first infection at approximately 5 months of age, when lambs express innate resistance. This selection should be based primarily on FEC, while also considering PCV and TPPr, given that the infection involves a hematophagous nematode.

## Data Availability

The data is available upon request to the authors.
